# Online Learning to Support Culturally Safe Communication with First Nations Australians in Radiation Therapy: A Pre-Post Intervention Study

**DOI:** 10.1177/10732748261423252

**Published:** 2026-03-06

**Authors:** L. Stoll, K. Carter, S. Jahan, H. M. Dhillon, J. Cunningham, S. Sabesan, G. K. B. Halkett, S. Baxi, G. Kar, J. Shaw, S. Smith, M. Penniment, A. Stoneley, L. McGhee, G. Garvey

**Affiliations:** 1 1974 First Nations Cancer & Wellbeing Research Program, School of Public Health, The University of Queensland, Brisbane, QLD, Australia; 2 Psycho-Oncology Cooperative Research Group, School of Psychology, Faculty of Science, 591258 The University of Sydney, Sydney, NSW, Australia; 3 Menzies School of Health Research, 2306 Charles Darwin University, Darwin, NT, Australia; 4 157842 Townsville Hospital and Health Service, Townsville, QLD, Australia; 5 1649 Curtin University, Perth, WA, Australia; 6 GenesisCare, Gold Coast, QLD, Australia; 7 Griffith University, Southport, QLD, Australia; 8 Alan Walker Cancer Care Centre, Darwin, NT, Australia; 9 7800 The University of New South Wales, Sydney, NSW, Australia; 10 1062 Royal Adelaide Hospital, Adelaide, SA, Australia; 11 95105 Icon Cancer Centre, South Brisbane, QLD, Australia

**Keywords:** communication skills training, indigenous, radiation therapy, online modules

## Abstract

**Introduction:**

Culturally safe communication is essential for supporting First Nations Australians undergoing radiation therapy. First Nations Australian patients, however, often face barriers in accessing culturally safe communication and cancer care. This study evaluates changes in healthcare professionals’ (HCPs) confidence, skills, and knowledge in culturally safe communication with First Nations cancer patients after completing an online learning program.

**Methods:**

This single group pre-post intervention study recruited HCPs from three regional Australian cancer centres. Pre- and post-training surveys, administered via Qualtrics, assessed self-reported confidence, knowledge, and communication skills for engaging with First Nations radiation therapy patients. Participants completed an online learning program consisting of three self-directed modules focused on cultural competency, health literacy and communication, and application of an Indigenous radiation therapy talking book resource. The post-training survey also included module evaluation items. Pre- and post-training data were analysed using paired-sample t-tests (α = 0.05). Descriptive statistics and content analysis were applied to evaluate participant feedback.

**Results:**

Of the 49 participants recruited, 38 participated in this study, most were non-Indigenous (94.7%), 52.6% were radiation therapists, and 65.8% reported seeing between 11-50 First Nations Australian patients annually. The participants’ mean confidence in communicating with First Nations Australian patients increased from 3.50 to 4.03 (*p* = 0.006), and preparedness to support patient needs rose from 3.55 to 3.95 (*p* = 0.04). The online modules were highly rated as good or excellent by 95% of participants.

**Conclusion:**

The findings demonstrate that tailored online learning modules can significantly enhance HCP’s self-reported confidence, skills, and knowledge in communicating with First Nations cancer patients receiving radiation therapy. Integrating training into routine practice may promote more culturally responsive cancer care, strengthening engagement and support for First Nations Australian radiation therapy patients.

## Plain Language Summary

**Why this research matters:** Culturally safe communication helps First Nations Australians feel respected, understood, and supported during cancer treatment. Yet, many First Nations people experience barriers to culturally safe care when receiving radiation therapy. To help improve this, our research evaluated an online training program for healthcare professionals (HCPs) in communicating well with First Nations Australians receiving radiation therapy.

**What we did:** We invited radiation therapy HCPs to complete three self-directed online learning modules. These modules focused on understanding culture, improving communication, and using a resource called the *Indigenous Radiation Therapy Talking Book* to support communication with patients. Participants completed surveys before and after the online learning program to see if their knowledge, confidence and skills in culturally safe communication improved.

**What we found:** Thirty-eight HCPs participated in this study. After completing the training, most reported feeling more confident, knowledgeable, and better prepared to communicate with First Nations patients. Almost all rated the modules as good or excellent.

**Why this is important:** Our findings show that an online training program designed to improve culturally safe communication can help HCPs to provide more respectful and responsive care. Implementation of this training program is likely to improve trust and communication between HCPs and First Nations Australians receiving radiation therapy.

## Background

Many people with cancer undergoing radiation therapy experience feelings of anxiety, worry, and uncertainty about the future and potential side effects, such as severe skin reactions, organ damage, or fatigue.^1^ Furthermore, stress and anxiety are commonly experienced by people diagnosed with cancer and subsequent treatment can further reduce their ability to process and act on health information.^2,3^

Effective communication during radiation therapy has the potential to play a critical role in addressing these concerns and supporting individuals throughout their treatment. For Aboriginal and Torres Strait Islander peoples (hereafter referred to as First Nations Australians), these concerns may be amplified by barriers to care, experiences of racism, cultural safety, and limited access to culturally appropriate information and services.^4,5^ Family, community, and connection to Country play a central role in supporting First Nations Australians throughout their cancer experience.^4,6^ Therefore, communication that is clear, respectful, and culturally safe is essential, not only between healthcare professionals (HCPs) and patients, but also family and community members.^7,8^ Such approaches can help build trust, reduce fear and uncertainty, and improve experiences and outcomes for First Nations Australians receiving radiation therapy.^7,8^ Therefore, enhancing the ability of healthcare services to provide culturally safe, high-quality care is a key strategy to improving cancer outcomes for First Nations Australians.

HCPs play a key role in addressing health literacy and health communication barriers to improving health outcomes for First Nations cancer patients.^7,9^ How HCPs communicate with patients influences how individuals understand, react to, and engage with their care and treatment.^7,10^ HCPs may not be aware of the cultural, emotional, or information needs and preferences of First Nations Australians with cancer, and individuals may be reluctant to raise concerns or disclose difficulties with understanding health information.^3,4,11^ In oncology settings, HCPs often report challenges explaining complex medical information and treatment procedures to First Nations Australians.^11^ Misunderstandings or misinterpretations may also arise due to sociocultural and linguistic differences, further compounding communication difficulties.^12^

Communication skills training (CST) can enhance positive treatment outcomes and improve patient-experiences.^13,14^ CST refers to evidence based educational programs designed to enhance HCPs abilities in communicating effectively with patients and families.^15^ Effective communication is especially important in oncology, where patients face the dual burden of processing a cancer diagnosis and navigating culturally specific challenges that may affect engagement with treatment.^7^ A cancer diagnosis is difficult in itself, and when combined with cultural and contextual concerns of First Nations Australians, poor communication by HCPs can result in reluctance to take up cancer treatment, ultimately leading to poorer cancer outcomes.^4,12^

Strengthening the capacity of health services to provide high-quality, culturally safe care for First Nations Australians affected by cancer is critical to improving cancer outcomes.^4^ However, there remains a lack of appropriate educational resources to support this goal.^16^ Existing programs are often limited to cultural awareness or cultural safety training and rarely provide practical, tailored strategies for effective communication.^4,12^ This study aimed to evaluate changes in confidence, skills, and knowledge of radiation HCPs participating in an online learning program developed to improve culturally safe communication with First Nations Australian cancer patients.

## Methods

### Study Overview

The study adheres to a quasi-experimental pre-post intervention design. The reporting of this study conforms to the Strengthening the Reporting of Observational Studies in Epidemiology (STROBE) Guidelines.^17^ This study is part of a larger project that focussed on improving patient-centred care and treatment outcomes for Aboriginal and Torres Strait Islander people through *Collaboration and Communication in Cancer Care’* (The 4C’s Project, GNT1152653). The 4Cs Project was led by a senior Aboriginal researcher, and First Nations Australian staff and community members were engaged in the development of the project outputs. These outputs included the development and evaluation of culturally appropriate strategies and resources for HCPs and First Nations cancer patients. An online learning program was specifically developed for HCPs to improve culturally safe communication between radiation HCPs, and First Nations Australians and is the focus of this publication.^18-20^ A new Indigenous Radiation Therapy Talking Book (IRTB)^21^ was designed for First Nations cancer patients, that could also be used by HCPs in discussions with patients about treatment options and during patient education sessions.^20,22^ The IRTB is available online in both English, and Yolgnu languages in both e-publication form or as a video playlist on YouTube, which can all be accessed on the dedicated PoCoG website page.^20^

### Ethics Approval

This study was conducted in accordance with the Declaration of Helsinki (revised 2024),^23^ and has received approval from the following Ethics Committees:

The 4C’s Project within which this study is an output, received primary ethical approval by the Northern Territory Department of Health and Menzies School of Health Research HREC (NTHREC; HREC ID: 2018-3177) on 24^th^ September 2018.

Once the primary research team transferred from the Menzies School of Health Research to The University of Queensland, external ethics approval was ratified by The University of Queensland HREC (UQHREC; Project ID: 2021/HE002475) on 11th November 2021.

Site specific ethics approvals were provided by the Far North Queensland HREC on the 25^th^ January 2019 (HREC/2018/QCH/46950), as well as the Townsville Hospital and Health Service HREC on the 20^th^ February 2019 (HREC/2018/QTHS/44459).

### Study Materials

#### Online Learning Program

The Online Learning Program comprised three modules: (1) Cancer Overview and Factors Impacting Health Inequalities; (2) Communicating with Patients and Carers; and (3) Using the Radiation Therapy Talking Book in Practice. The modules were co-developed by the Psycho-Oncology Co-operative Research Group (PoCoG) in collaboration with researchers from an Aboriginal-led Centre of Research Excellence in Targeted Approaches to Improve Cancer Services for Aboriginal and Torres Strait Islander Australians (TACTICS CRE).^24^ Module design was informed by existing literature,^7,25-33^ the Indigenous Allied Health Australia Framework,^34^ national policy documents and cancer care guidelines on culturally safe communication with First Nations Australians,^35,36^ and guided by adult-learning principles. Each self-directed e-module was delivered in an audio-visual format. Each module began with an Acknowledgement of Country and incorporated narration by a First Nations Australian actor to promote cultural safety and alignment with best practices in Indigenous health and education.^24^ Participants were expected to complete all three modules, totalling approximately 54 minutes, at their own pace, with completion tracked via the Qualtrics platform.^37^ A summary of the modules and their respective learning objectives and topics covered are outlined in Table 1. Modules 1 and 2 are available online on YouTube^18,19^ and on the PoCoG website,^20^ whilst Module 3 is currently unpublished.

**Table 1.** Summary of the Online Learning Program Modules

	Learning objectives	Topics covered
Module 1: Cancer overview and factors impacting health inequalities^18^ (23 minutes)	After completion of Module 1, participants will have a greater understanding of:	• Cancer burden among First Nations Australians
	• First Nations Australians’ health and the factors that contribute to the health inequality gap	• Inequities across the cancer continuum
	• The cancer indicators and trends for First Nations Australians	• Influence of individual, service, and system-level factors on outcomes
	• Some of the factors that impact cancer outcomes for First Nations Australians	• Enablers of improved care: cultural safety, effective communication, access to appropriate resources
		• Featured a First Nations Australian survivor’s reflections on lived experiences of cancer diagnosis, treatment, and survivorship
Module 2: Communicating with patients and carers^19^ (21 minutes)	After completion of Module 2, participants will be able to:	• Introduced health literacy and factors influencing understanding among First Nations Australians
	• Understand the concepts of ‘health literacy’ and ‘patient-centred care’ as relevant to First Nations Australian cancer patients	• Outline of strategies for HCPs: To build rapport; create safe environments; recognise their impact; consider cultural complexities; draw on supports; and adapt communication approaches when needed
	• Understand the significance of worldview and culture in experiencing health and healthcare	
	• Identify differences in communication approaches	
	• Understand ways to optimise communication interactions	
Module 3: Using the Radiation Therapy Talking Book in Practice^20-22^ (10 minutes)	After completion of Module 3, participants will:	• Overview of what is a talking book and its purpose in patient education
	• Understand the purpose of Talking Books	• Examples of the benefit of a low literacy talking book in practice for non-Indigenous patients
	• Recognise how and why the 4Cs project developed the IRTB	• Introduction and overview of the IRTB developed by the 4Cs project
	• Learn how to use the IRTB in clinical practice with First Nations Australian patients	• When and how to use the IRTB in practice

### Participants and Recruitment

**Figure 1.**
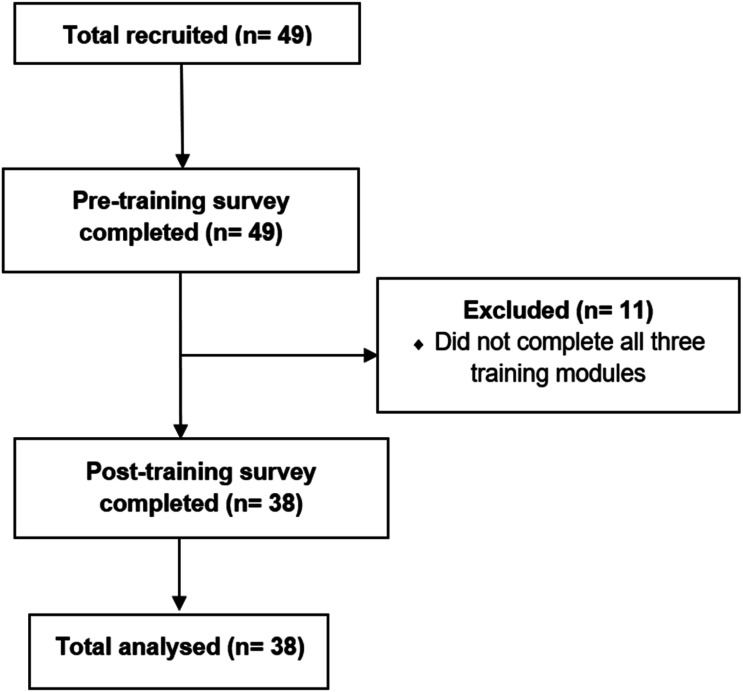
Participant flow chart

Eligible participants included HCPs (radiation therapists, nurses, doctors, Aboriginal Liaison Officers) from the three participating regional cancer care centres, two of which are in Northern Queensland and one in the Northern Territory, Australia. These centres were chosen because they serve large numbers of First Nations patients living in regional and remote locations, reflect diverse regional service delivery contexts, and provide an opportunity to assess HCP training needs in culturally safe cancer care. Participants were therefore recruited using convenience sampling with a rolling enrolment across the study period (May 2023 to July 2024). Participants were informed about the study via email from Department Heads and the local site study manager. Recruitment occurred on a rolling basis, as sites identified HCPs to participate in training, potentially reducing selection bias. The final analytic sample included 38 participants who completed both pre- and post-training surveys, reducing attrition bias. No formal a priori power calculation was conducted, as this study was an exploratory evaluation of the immediate impact of the online learning modules on HCPs’ self-reported communication skills, knowledge, and confidence. The sample size reflects the number of HCPs available and willing to participate across the three regional centres, which was inherently limited by the size of the workforce at these sites (Figure 1).

### Procedure

HCPs were provided with a Qualtrics^37^ platform study link to access information about the study, and if they agreed to participate, they provided written, informed consent online. Participants’ privacy was protected through a deidentification process, removing any identifiable data (name, email, address) upon exporting the raw Qualtrics data. Each participant was assigned a unique study identification code. All data was securely stored on the University of Queensland’s Research Data Manager (RDM) platform, a secure cloud-based storage system accessible only to research team members responsible for data management and analysis.

The pre- and post-training survey items were informed by existing literature,^7,25-33^ the Indigenous Allied Health Australia Framework,^34^ national policy documents and cancer care guidelines on culturally safe communication with First Nations Australians.^35,36^ The pre- and post-training surveys were developed in collaboration with the PoCoG Research group and researchers from the Aboriginal-led TACTICS CRE. Although no formal psychometric evaluation was conducted, internal reliability of the six confidence, skills, and knowledge items were assessed, with Cronbach’s α = 0.81. The full self-reported Confidence, Skills, and Knowledge questionnaire, and Comments and Feedback questionnaire are provided in Supplemental Tables 1A and 1B.

Prior to completing the online modules, participants completed a pre-training survey (T1) that included questions about the participants’ professional background and five Likert scale questions (1-Strongly disagree to 5-Strongly agree), assessing self-reported confidence, skills and knowledge regarding First Nations Australians’ needs, health context, cultural competency, communication and support abilities, and effect of cultural knowledge on care provided (Supplemental Table 1A).

The training component required participants to view the three online learning modules uploaded on the Qualtrics platform. Participants could view the modules in their own time, with the opportunity to go back and review content at any time.

Following completion of the three online learning modules, participants completed a post-training survey (T2), which repeated the questions to reassess HCPs self-reported confidence, skills and knowledge. They were also asked to rate the training modules overall, including clarity of objectives, relevance of content, usefulness to practice, and ease of following, using four Likert scale questions (1 = Very poor to 5 = Excellent, or 1 = Strongly disagree to 5 = Strongly agree). The survey concluded with three open-ended questions, inviting participants to provide feedback and suggestions for improvement of the modules (Supplemental Table 1B).

### Analyses

SPSS Statistical software was used for data analysis.^38^ Descriptive statistics (means, standard deviations, frequencies) were calculated for participant characteristics and survey responses. Paired-sample t-tests were conducted to explore changes in HCPs’ self-reported confidence, skills, and knowledge pre- and post-training, with statistical significance set at *p* < 0.05. Normality of difference scores and potential outliers were assessed through visual inspection of histograms and boxplots. To account for multiple comparisons across six outcomes, Holm-adjusted *p*-values were calculated. A significance threshold of α = 0.05 was applied sequentially according to the Holm procedure and reported.

Effect sizes (Cohen’s d) with 95% confidence intervals were calculated for each outcome to quantify the magnitude of observed changes. Internal consistency reliability of the six-item survey scale was assessed using Cronbach’s α. Missing data were handled listwise, and all available pre- and post-training responses were included in the analyses.

For program evaluation, HCPs’ response ratings of “agree” or “strongly agree” were considered to indicate HCPs satisfaction with the modules, and a descriptive summary of open-text feedback was used to assess the key enablers and barriers of the online learning program.

## Results

### Participants

Of the 49 participants recruited, 38 completed all three modules and the pre-post training survey. Most identified as non-Indigenous (n = 36, 94.7%). Over half of the participants were radiation therapists (n = 20, 53%), followed by radiation oncologists (n = 8, 21%), clinical nurses (n = 6, 16%), and other (n = 4, 10.5%). Most participants reported seeing between 11-50 First Nations Australian patients annually (n = 25, 66%) while 12 participants (32%) reported seeing over 50 First Nations Australian patients annually.

### HCP Confidence, Skills, and Knowledge

Overall, HCPs’ self-reported levels of confidence, skills, and knowledge significantly increased following completion of the online learning modules (Table 2). After Holm correction, HCPs reported feeling significantly better equipped to communicate with First Nations Australians about cancer and cancer treatment following training (mean increase = 0.53, *p* = 0.006). Similarly, they reported feeling better prepared to support the needs of First Nations Australians following the training (mean increase = 0.4, *p* = 0.04). HCPs’ knowledge about the specific needs of First Nations Australian patients influencing care also improved following the training (mean increase = 0.34, *p* = 0.01). No significant changes were observed in self-reported understanding of the factors contributing to First Nations Australians’ health outcomes, how First Nations Australians may experience healthcare differently to non-First Nations Australians or knowledge of whom to approach to ensure First Nations Australians receive culturally competent support.

**Table 2.** Changes in HCPs’ Self-Reported Confidence, Skills, and Knowledge Pre- and Post-training (N = 38)

Item	Pre mean (±SD)	Post mean (±SD)	Mean diff (SD)	t (df = 37)^c^	Holm-adjusted *p*	Cohen’s d (95% CI)^d^
To what extent does your knowledge about the specific needs about Aboriginal and Torres Strait Islander patients influence how you provide care during radiation therapy treatment?^a^	3.34 (0.58)	3.68 (0.47)	0.34 (0.63)	−3.36	0.012	0.63 (0.25-1.01)
I have a good understanding of the factors which contribute to the health outcomes of Aboriginal and Torres Strait Islander peoples.^b^	3.82 (0.73)	4.13 (0.78)	0.32 (0.81)	−2.41	0.063	0.81 (0.30-1.32)
I have a good understanding of the factors which can contribute to Aboriginal and Torres Strait Islander peoples experiencing healthcare settings differently to non-First Nations Australians.^b^	3.79 (0.84)	4.11 (0.80)	0.32 (0.93)	−2.09	0.088	0.93 (0.09-1.77)
I know who I can approach to ensure Aboriginal and Torres Strait Islander patients receive culturally competent support.^b^	4.08 (0.91)	4.24 (0.85)	0.16 (1.03)	−0.95	0.35	1.03 (−0.20-2.26)
I feel well equipped to communicate with Aboriginal and Torres Strait Islander peoples about cancer and cancer treatment.^b^	3.50 (0.76)	4.03 (0.75)	0.53 (0.92)	−3.52	0.006	0.92 (0.38-1.46)
I feel well prepared to support the needs of Aboriginal and Torres Strait Islander cancer patients.^b^	3.55 (0.76)	3.95 (0.80)	0.40 (0.92)	−2.65	0.036	0.81 (0.25-1.37)

Missing responses n = 0

^a^Utilised scoring of 1, Not at all; 2, A little; 3, Moderately; 4, A lot

^b^Utilised scoring of 1, Strongly disagree; 2, Disagree; 3, Neutral; 4, Agree; 5, Strongly agree

^c^Negative *t*-values indicate higher post-training scores.

^d^Effect sizes interpreted as small (0.2), moderate (0.5), and large (0.8).

### Feedback and Evaluation on the Online Learning Program

Overall, the training modules were positively received, with 95% of participants rating them as good or excellent. Participants provided very positive feedback, with 92% of participants agreeing or strongly agreeing the objectives were clearly defined; 100% the topics were relevant to their practice; 97% that content was organised and easy to follow; and 100% agreed the training would be useful in their work (Supplemental Table 2A).

Most participants provided positive feedback open-text responses (n = 31, 82%). Supplemental Table 2B provides a summary of the feedback provided by the participants for the online learning modules. Participants highlighted particularly valuing the audiovisual format and delivery of the modules, stating they were informative, engaging, and concise; as well as the highlighting the relevance and usefulness of the practical communication tools provided. Half of the participants provided constructive feedback through the open-text responses (n = 19, 50%). Key points of constructive feedback provided included: wanting more practical examples of communication tools and approaches; wanting more interactivity and access to the learning resources/slides; and that the audiovisual content presented be more concise in length and detail.

## Discussion

This study evaluated the impact of a novel online learning program on the self-reported knowledge, skills, and confidence levels of radiation therapy HCPs providing care to First Nations Australians. The modules were well-received and resulted in significant improvements in HCPs’ self-reported confidence, skills, and knowledge regarding culturally safe care. These findings align with previous studies of non-Indigenous CST, which demonstrated similar improvements in HCPs’ self-reported competencies following training.^15,39-42^

Following completion of the modules, HCPs reported significant improvements across multiple areas, including knowledge of First Nations Australians’ specific needs, understanding factors contributing to health outcomes, and confidence in communicating about cancer and treatment. Participants also felt better prepared to support the needs of First Nations Australians. Knowing whom to approach for culturally competent support showed little change, likely due to high baseline scores, and reflecting pre-existing awareness, which could be largely due to their reports of seeing many First Nations patients in their service. Overall, these findings indicate the program effectively reinforced existing knowledge, while addressing gaps in confidence and practical skills. These findings are consistent with prior studies on cultural safety and communication skills training, which have reported increases in HCPs’ self-reported knowledge, confidence, and skills following targeted interventions.^15,39-42^

While improvements in HCPs’ self-reported knowledge and skills are encouraging, effective culturally safe care also depends on broader systemic and workforce factors. The study sites serve a large number of First Nations Australian people from rural, remote, and very remote parts of Australia, who are at a higher risk of low health literacy and increased communication barriers.^12^ Additionally, the study included very few First Nations Australian HCPs, reflecting a national workforce disparity: only 1.2% of registered HCPs across all regulated professions identify as First Nations Australian.^43^ Aboriginal health workers (AHWs) have demonstrated improvements in patient outcomes in their communities by facilitating culturally competent care, and bridging gaps in communication.^44^ This highlights the importance of continuing to engage non-Indigenous radiation HCPs through training, while simultaneously creating pathways and incentives to increase First Nations Australian representation within the radiation oncology workforce.

Providing Indigenous specific CST for radiation HCPs is a crucial step towards being able to provide culturally safe healthcare through effective health communication for First Nations Australian cancer patients and their families.^5,45^ Although the online learning program showed significant gains in HCPs’ self-reported knowledge, confidence, and skill, we did not investigate if these gains translated into practice changes or improved patient outcomes. The Centre for Cultural Competence Australia emphasises that cultural awareness is not enough; organisations must demonstrate cultural competency through behavioural change and measurable improvements in patient care.^46^ Similar to other studies, relying on self-reported outcomes,^40,41^ there is limited evidence on the long-term retention of knowledge and skills or their impact on patient satisfaction. Future research should examine First Nations Australian patient experiences, patient reported outcomes and satisfaction with care delivered by HCPs who have completed the training.

Despite significant improvements in HCPs’ knowledge, skills, and confidence after completion of the online learning modules, this study has some limitations that should be acknowledged. This study used a pre–post design without a control group, which may introduce potential biases such as history effects (external events influencing participants during the study period), maturation (natural changes over time), and the Hawthorne effect (participants modifying behaviour because they are being observed), limiting the ability to attribute observed changes solely to the training. However, as the post-training survey was completed on the same day or within one week after training, these factors are less likely to have substantially influenced the findings and if present, would most likely have slightly overestimated the observed effects. The sample size was relatively small, limiting subgroup analyses by professional role and the generalisability of findings to the broader HCP workforce, particularly Indigenous HCPs. The outcomes were self-perceived, so, as noted above, it is unclear whether the observed improvements translated into actual changes in practice or patient outcomes. Additionally, the study assessed immediate post-training effects only, precluding evaluation of long-term retention or communication changes. Finally, the low representation of First Nations Australian HCPs reflects broader workforce disparities and limits insights into the perspectives of Indigenous HCPs.

## Conclusion

This study demonstrates that an Indigenous specific online communication training program can significantly improve HCPs’ self-reported knowledge, confidence, and skills in providing culturally safe care to First Nations Australians. Participants valued the audiovisual content, First Nations Australian narrators, survivor stories, and practical communication tools, and suggested enhancements such as interactive role-plays and additional patient perspectives.

These findings suggest such online programs should be part of strategies to strengthen culturally safe care. Broader implementation could support HCPs in improving communication and cancer care outcomes for First Nations Australians. Future research should assess whether these gains translate into clinical practice improvements, patient satisfaction, and long-term retention of knowledge and skills.

## Supplemental Material

Supplemental Material - Online Learning to Support Culturally Safe Communication with First Nations Australians in Radiation Therapy: A Pre-Post Intervention Study Supplemental Material for Online Learning to Support Culturally Safe Communication with First Nations Australians in Radiation Therapy: A Pre-Post Intervention Study by L. Stoll, K. Carter, S. Jahan, H. M. Dhillon, J. Cunningham, S. Sabesan, G. K. B. Halkett, S. Baxi, G. Kar, J. Shaw, S. Smith, M. Penniment, A. Stoneley, L. McGhee, G. Garvey in Cancer Control.

## Data Availability

The data underlying this article is not available due to ethical and privacy restrictions.*

## Appendix

### Acronyms

CST: Communication skills training
HCPs: Healthcare professionals.
