# Supplementary material for: Online Learning to Support Culturally Safe Communication with First Nations Australians in Radiation Therapy: A Pre-Post Intervention Study
Source: Cancer Control. 2026 Mar 6;33:10732748261423252. doi: 10.1177/10732748261423252 (PMC12966548; doi:10.1177/10732748261423252)
Supplement: Supplemental Material - Online Learning to Support Culturally Safe Communication with First Nations Australians in Radiation Therapy: A Pre-Post Intervention Study [file sj-pdf-1-ccx-10.1177_10732748261423252.pdf]

Appendices

Supplementary Table 1A: Confidence, Skills and Knowledge Questions

To what extent does your knowledge about the specific needs about Aboriginal and Torres Strait Islander patients influence how you provide care during radiation therapy treatment?

Not at all	A little	Moderately	A lot
○	○	○	○

Please indicate to what extent you agree with the following statements.

	Strongly disagree	Disagree	Neutral	Agree	Strongly agree
I have a good understanding of the factors which contribute to the health outcomes of Aboriginal and Torres Strait Islander peoples.	○	○	○	○	○
I have a good understanding of the factors which can contribute to Aboriginal and Torres Strait Islander peoples experiencing healthcare settings differently to non-First Nations Australians.	○	○	○	○	○
I know who I can approach to ensure Aboriginal and Torres Strait Islander patients receive culturally competent support.	○	○	○	○	○
I feel well equipped to communicate with Aboriginal and Torres Strait Islander peoples about cancer and cancer treatment.	○	○	○	○	○
I feel well prepared to support the needs of Aboriginal and Torres Strait Islander cancer patients.	○	○	○	○	○

Supplementary Table 1B: Comments and Feedback Questions

How do you rate the training overall?

Very poor	Poor	Average	Good	Excellent
○	○	○	○	○

Please indicate your impressions of the items listed below:

	Strongly disagree	Disagree	Neutral	Agree	Strongly agree
The objectives of the training were clearly defined.	○	○	○	○	○
The topics covered were relevant to me.	○	○	○	○	○
The content was organized and easy to follow.	○	○	○	○	○
This training experience will be useful in my work.	○	○	○	○	○

What did you like most about this training?

What aspects of the training could be improved?

Supplementary Table 2A: HCPs' Ratings of the Online Learning Program (n= 38)

	Neutral (%)	Agree (%)	Strongly agree (%)	Total agree or strongly agree (%)
*How do you rate the training overall?**	2 (5.3)	24 (63.2)	12 (31.6)	36 (94.8)
The objectives of the training were clearly defined	3 (7.9)	22 (57.9)	13 (34.2)	35 (92.1)
The topics covered were relevant to me	0 (0)	18 (47.4)	20 (52.6)	38 (100)
The content was organized and easy to follow	1 (2.6)	19 (50)	18 (47.4)	37 (97.4)
This training experience will be useful in my work	0 (0)	21 (55.3)	17 (44.7)	38 (100)

**For the first item, response options were average, good, and excellent (equivalent to neutral, agree, strongly agree)*

Supplementary Table 2B: Descriptive summary of participant feedback on Online Learning Program

**What did you like most about this training? (n= 31)**	
**Coded Themes**	**Participant responses**
Relevance and usefulness in culturally appropriate communication	• *"…The video from a patient's perspective was informative."*<br>• *"…I also liked that there were specific Radiation Therapy targeted parts."*<br>• *"Very informative and relevant"*<br>• *"The explanation of the use of silence in conversations is really useful and something that I will happily use myself and value when someone else gives it to me."*<br>• *"Explaining communication techniques"*<br>• *"Excellent breakdown of all factors and strategies for developing communication skills. Now I have a new tool to try out in my Radiotherapy education to Aboriginal people."*<br>• *"Relevant and up-to-date, with upfront and honest mention of limitations and 9es from both clinician and patient perspective."*

	• *"It will provide addition support and tools to communicate with Aboriginal and Torres Strait Islander patients."*
Preference of audio-visual delivery	• *"The artwork and visuals were also very good."*<br>• *"Video presentation"*<br>• *"The video was easier to take in rather than reading for long periods of time, and it was reasonably quick…"*<br>• *"Audio-visual tools. In depth but not overwhelming presentation"*<br>• *"Videos are a great format"*<br>• *"watching videos was a great form of education and listening a story from a cancer patient"*
**What aspects of the training could be improved? (n= 19)**	
**Coded Themes**	**Participant responses**
More practical examples of communication	• *"More patient experiences would be helpful, especially things they found useful/not useful from staff"*<br>• *"Maybe some more examples of using these tools"*<br>• *"Perhaps a video of someone actually providing radiotherapy education to an Aboriginal person"*<br>• *"Perhaps more practical advice for advising best approach"*<br>• *"Training could include a video of a patient being communicated to more practically demonstrate."*
Interactivity and access to learning resources	• *"More interactive"*<br>• *"Sample pages of the talking book"*<br>• *"Summaries of the topics in writing that was discussed in the videos"*<br>• *"Some interactive learning could be helpful"*<br>• *"Interaction with information presented"*<br>• *"Access to presentation slides"*
Length and detail of training	• *"Length of videos are not ideal to fit into a busy schedule"*<br>• *"Make videos more concise"*<br>• *"Slides were good but sometimes busy, I kept on looking where I was to marry up the voice over with the slide content"*<br>• *"There was some parts that I found quite wordy and hard to follow"*
